# Association of common genetic variants related to atrial fibrillation and the risk of ventricular fibrillation in the setting of first ST-elevation myocardial infarction

**DOI:** 10.1186/s12881-017-0497-1

**Published:** 2017-11-21

**Authors:** Reza Jabbari, Javad Jabbari, Charlotte Glinge, Bjarke Risgaard, Stefan Sattler, Bo Gregers Winkel, Christian Juhl Terkelsen, Hans-Henrik Tilsted, Lisette Okkels Jensen, Mikkel Hougaard, Stig Haunsø, Thomas Engstrøm, Christine M. Albert, Jacob Tfelt-Hansen

**Affiliations:** 1 Department of Cardiology, Rigshospitalet, Copenhagen University Hospital, Blegdamsvej 9, 2100 Copenhagen Ø, Denmark; 2 Department of Cardiology, Aarhus University Hospital, Skejby, Nørrebrogade, 44, 8000 Aarhus C, Denmark; 3 Department of Cardiology, Aalborg University Hospital, Hobrovej 18-22, 9100 Aalborg, Denmark; 4 Department of Cardiology, Odense University Hospital, Søndre Blvd. 29, 5000 Odense C, Denmark; 5 Laboratory of Molecular Cardiology, Department of Cardiology, Copenhagen University Hospital Rigshospitalet, Juliane Mariesvej 20, 2100 Copenhagen Ø, Denmark; 6 Center for Arrhythmia Prevention, Division of Preventive Medicine, Cardiovascular Division, Department of Medicine, Brigham and Women’s Hospital, Harvard Medical School, 75 Francis Street, Boston, MA 02115 USA; 7 Department of Cardiology, University of Lund, Lund, Sweden

**Keywords:** Ventricular fibrillation, Atrial fibrillation, Myocardial infarction, ST-elevation myocardial infarction, Sudden cardiac death, Genetics, Single nucleotide polymorphisms

## Abstract

**Background:**

Cohort studies have revealed an increased risk for ventricular fibrillation (VF) and sudden cardiac death (SCD) in patients with atrial fibrillation (AF). In this study, we hypothesized that single nucleotide polymorphisms (SNP) previously associated with AF may be associated with the risk of VF caused by first ST-segment elevation myocardial infarction (STEMI).

**Methods:**

We investigated association of 24 AF-associated SNPs with VF in the prospectively assembled case–control study among first STEMI-patients of Danish ancestry.

**Results:**

We included 257 cases (STEMI with VF) and 537 controls (STEMI without VF). The median age at index infarction was 60 years for the cases and 61 years for the controls (*p* = 0.100). Compared to the control group, the case group was more likely to be male (86% vs. 75%, *p* = 0.001), have a history of AF (7% vs. 2%, *p* = 0.006) or hypercholesterolemia (39% vs. 31%, *p* = 0.023), and a family history of sudden death (40% vs. 25%, *p* < 0.001). All 24 selected SNPs have previously been associated with AF. None of the 24 SNPs were associated with the risk of VF after adjustment for age and sex under additive genetic model of inheritance in the logistic regression model.

**Conclusion:**

In this study, we found that the 24 AF-associated SNPs may not be involved in increasing the risk of VF. Larger VF cohorts and use of new next generation sequencing and epigenetic may in future identify additional AF and VF risk loci and improve our understanding of genetic pathways behind the two arrhythmias.

## Background

Cohort studies have revealed an increased risk for ventricular fibrillation (VF) [1–4] and sudden cardiac death (SCD) [5–7] in patients with atrial fibrillation (AF). In the Danish GEVAMI (**GE**netic causes of **V**entricular **A**rrhythmias in patients with first ST-elevation **M**yocardial **I**nfarction) study we have previously demonstrated a strong association between AF and VF before primary percutaneous coronary intervention (PPCI) with an odds ratio (OR) of 2.8 (95% CI: 1.10–7.30) [1]. The GEVAMI study population includes patients presenting with first ST-segment elevation myocardial infarction (STEMI) that do (case) or do not (control) develop VF prior to primary angioplasty.

Heritable factors and cardiac channelopathies have been suggested as cause of VF and AF [8, 9]. Genome-wide association studies (GWAS) in individuals of European, [10, 11] Asian, [12] and African American [13] descent have identified several genomic regions with AF. Especially single nucleotide polymorphisms (SNP) on chromosomes 4q25 (*PITX2*) have strong association with AF [9, 14], but also with higher risk of SCD [15]. Furthermore, *SCN5A* and *SCN10A* which encode the transient sodium channel (I_Na_) and play a pivotal role in membrane depolarization during cardiac action potentials have also been associated with AF, [16, 17] VF [17], and also Brugada Syndrome [18, 19].

In this study, we hypothesized SNPs that previously were associated with AF can affect the risk of VF in the GEVAMI study population and thereby suggest a shared genetic pathway.

## Methods

### Study population

The study population and design has been described previously [1, 20]. In brief, the Danish GEVAMI study is an ongoing nationwide prospectively collected case-control study among patients with first STEMI between the ages of 18 and 80 years [1, 20]. The case group are STEMI patients who had VF or sustained ventricular tachycardia (VT; *n* = 6) with cardiac arrest within the first 12 h of symptoms of STEMI before PPCI, and the control group are STEMI patients who did not have VF/VT. All the patients are collected at all four PPCI centers in Denmark. Both groups were required to have cardiac symptoms lasting ≤12 h, acute STEMI on ECG, which required acute PPCI. Baseline demographics and previous medical history are collected by research coordinators utilizing pre-designed questionnaires and whole blood is collected for genetic analysis.

### DNA extraction and SNP genotyping

The methods for DNA extraction and also the SNP genotyping and analysis of this study have been described previously in detail [20]. In brief, we used whole blood and isolated the total genomic DNA using LGC’s Kleargene™ silica-based DNA extraction technique, which was performed at LGC Genomics. Isolated DNA was then analyzed using UV spectrophotometry to estimate both the quality and quantity of the DNA and normalized.

Based upon the results of previous genetic association studies, 24 common genetic variants known to be associate with AF [9–12, 14, 16, 17, 21–27] were selected for SNP genotyping in both the cases and controls. SNP genotyping was performed using KASP™ genotyping assays from LGC genomics (http://www.lgcgenomics.com). For each SNP two allele specific forward primers and one reverse primer were designed (LGC Genomics, Hoddesdon, UK) as described in detail previously [20]. All assays were conducted without any knowledge of case or control status. Genotypes for all SNPs passed our quality-control threshold (call-rate ≥ 94%; Hardy-Weinberg equilibrium *P* > 0.05 in control subjects).

### Statistical analysis

Medians or proportions of baseline and presenting characteristics were computed for cases and controls, and significance of associations were tested using the Wilcoxon rank-sum test for continuous variables and the χ2 test or Fisher exact test (where appropriate) for categorical variables. A two-tailed *p* value ≤0.05 was considered statistically significant. A logistic regression model were constructed to estimate OR for the association between each SNP and VF using an additive model (per copy allele frequency) of inheritance, as described previously [20]. The logistic regression model was adjusted for age and sex. All analyses were performed using the Stata software package version 12.0 (StataCorp).

## Results

### Clinical characteristics of the cohort

The clinical characteristics of the cohort are described previously [1, 20]. In total, 257 cases (35 women and 222 men) with VF caused by first STEMI and 537 STEMI controls (131 women and 426 men) who did not developed VF were included in this prospective assembled study. Table 1 shows the baseline characteristics of the GEVAMI cohort. The median age of the cases was 60 (interquartile range (IQR); 53–68) years versus 61 (IQR; 52–66) years for the controls. As reported previously, compared to the controls, cases were more likely to be male, have atrial fibrillation or hypercholesterolemia, and a family history of sudden death [1]. The statin therapy was higher in the cases compared to the controls, most likely due to the higher degree of hypercholesterolemia in the cases. Levels of average weekly alcohol intake were higher in the cases (6 units/week) compared to the controls (3 units/week) (*P* = 0.001). Regarding other cardiovascular risk factors such as smoking, diabetes, and hypertension, did cases not differ significantly from the controls.

**Table 1:** Baseline characteristics of the cohort

Variables	Cases (*n* = 257)	Controls (*n* = 537)	*P* value
Female sex, No. (%)	35 (14)	131 (25)	0.001
Median Age at index infarction, y (IQR)	60 (53–68)	61 (52–66)	0.100
Cardiovascular risk profile			
Body mass index (kg/m^2^), (IQR)	27.2 (25–29)	26.7 (24–29)	0.400
Smoking (pack year), (IQR)	25 (5–41)	25 (6–42)	0.200
Smoking, No. (%)			
Never	38 (16)	108 (20)	0.300
Past	69 (28)	133 (25)	
Current	136 (56)	290 (55)	
Alcohol per week, (unit*, IQR)	6 (1–15)	3 (0–9)	<0.001
Alcohol units per week (categorized), No. (%)			
Non-drinkers	46 (19)	143 (27)	<0.001
Normal (1–7)	90 (38)	242 (46)	
Moderate High (8–14)	41 (17)	70 (13)	
High (>15)	60 (26)	73 (14)	
Diabetes, No. (%)	30 (12)	47 (9)	0.200
Hypertension, No. (%)	102 (41)	184 (35)	0.070
COPD, No. (%)	12 (5)	30 (6)	0.700
Hypercholesterolemia, No. (%)	97 (39)	165 (31)	0.023
Stroke, No. (%)	18 (7)	26 (5)	0.200
Atrial fibrillation, No. (%)	16 (7)	10 (2)	0.006
Depression, No. (%)	28 (11)	65 (12)	0.700
Epilepsy, No. (%)	4 (2)	5 (1)	0.500
Family History, No. (%)			
Sudden death	94 (40%)	128 (25%)	<0.001
Myocardial infarction	90 (40%)	195 (38%)	0.600
Stroke	36 (16%)	75 (15%)	0.600
Medication before MI^¶^, No. (%)			
β-blockers	20 (8)	43 (8)	0.900
Statins	55 (22)	65 (12)	<0.001
ACE/ARB blockers	50 (21)	90 (17)	0.200
Aspirin	28 (12)	40 (8)	0.060

IQR: interquartile range; unit^‡^ of alcohol =12 g (1 drink); COPD: chronic obstructive pulmonary disease; MI: myocardial infarction; ACE/ARB: angiotensin-converting-enzyme inhibitor/ angiotensin II receptor blocker

### Common genetic variants associated with VF

Comprehensive panels of 24 common variants were genotyped in 794 individuals in the GEVAMI population with Danish descent (Table 2). All the selected SNPs were reported to be associated with AF. The overall call rate was ≥95%. Table 2 shows the risk allele frequencies and the OR for each SNP. The rs6795970 SNP risk allele A did not exist in our cohort although the average call-rate was 97.8%. None of the 24 common genetic variants were associated with the risk of VF in the GEVAMI population after multivariable adjustment (Table 2).

**Table 2:** Additive genetic model of inheritance (per copy allele frequency) for association of 24 SNPs previously associated with atrial fibrillation, and in this study investigated for association with ventricular fibrillation before ST-segment elevation myocardial infarction

	Locus	SNP	RA	RAF (Cases/Controls)	OR	95% CI	P	Nearest gene symbol
1&	1q21	rs6666258	C	0.31/0.33	0.91	0.72–1.15	0.400	*KCNN3* [9, 26]
2	1q24	rs3903239	C	0.46/0.49	0.85	0.68–1.07	0.200	*PRRX1* [9]
3	3p25	rs4642101	G	0.61/0.62	0.99	0.79–1.25	0.900	*CAND2* [22]
4#*	3p21	rs6795970	A	0.00/0.00	–	–	–	*SCN10A* [17]
5&*	4q25	rs2200733	T	0.08/0.08	0.96	0.65–1.42	0.900	*PITX2* [9, 10]
6&	4q25	rs2634073	A	0.14/0.15	0.88	0.65–1.20	0.400	*PITX2* [9]
7&	4q25	rs6843082	G	0.16/0.18	0.87	0.66–1.17	0.400	*PITX2* [9, 14]
8	4q25	rs1448818	G	0.26/0.24	1.13	0.89–1.43	0.300	*PITX2* [21]
9&	4q25	rs10033464	T	0.76/0.94	0.79	0.53–1.17	0.300	*PITX2* [21, 27]
10	4q25	rs17570669	T	0.06/0.08	0.74	0.48–1.12	0.200	*PITX2* [21]
11	4q25	rs2723288	T	0.29/0.28	1.08	0.86–1.35	0.500	*PITX2* [21]
12	4q25	rs4400058	A	0.08/0.09	0.80	0.54–1.18	0.300	*PITX2* [21]
13	4q25	rs6838973	T	0.43/0.44	0.95	0.76–1.18	0.700	*PITX2* [21]
14	4q25	rs3853445	C	0.26/0.25	0.99	0.78–1.27	0.900	*PITX2* [21]
15	5q31	rs2040862	T	0.18/0.16	1.15	0.87–1.53	0.300	*PITX2* [21]
16	6q22	rs13216675	T	0.68/0.69	0.95	0.75–1.18	0.600	*GJA1* [22]
17	7q31	rs3807989	G	0.41/0.42	1.00	0.81–1.25	0.900	*CAV1* [9]
18	9q22	rs10821415	A	0.39/0.39	0.97	0.78–1.20	0.800	*C9ORF3* [9]
19	10q22	rs10824026	G	0.13/0.15	0.85	0.62–1.16	0.300	*MYOZ1* [9]
20	10q24	rs12415501	T	0.15/0.16	0.92	0.68–1.23	0.600	*NEURL* [22]
21	12q24	rs10507248	T	0.75/0.74	1.05	0.82–1.33	0.700	*TBX5* [22]
22	14q23	rs1152591	T	0.51/0.48	1.11	0.89–1.38	0.300	*SYNE2* [9]
23	15q24	rs7164883	G	0.17/0.13	1.28	0.96–1.70	0.090	*HCN4* [9]
24	16q22	rs2106261	A	0.18/0.17	1.09	0.83–1.44	0.500	*ZFHX3* [9, 36]

SNP: single-nucleotide polymorphism; RA: Risk allele; RAF: Risk allele frequency in cases over controls in our cohort; OR: odds ratio; CI: confidence interval; P value for the additive genetic model of inheritance (per copy allele frequency)

**#**: No risk allele (A) exists in the GEVAMI cohort. Logistic regression models under an additive model of inheritance adjusted for age and sex. Number of cases = 257; number of controls = 537

**&**: The SNP rs6666258 is in linkage disequilibrium (r^2^ = 1.00) with rs13376333

**&**: The SNP 2200733 is in linkage disequilibrium (r^2^ = 1.00) with rs6817105 and rs17042171

**&**: The rs2634073 is in linkage disequilibrium (r^2^ = 0.80) with rs6843082

**&**: The rs10033464 is in linkage disequilibrium (r^2^ = 1.00) with rs4032974

*: The SNP rs6795970 and 2,200,733 are both associated with SCD [20]

## Discussion

In this study, we investigated the role of 24 common SNPs previously associated with AF and their associations with VF in the setting of first STEMI and found no associations in the GEVAMI case-control population. To support the idea behind the hypothesis of this study several cohort studies including our GEVAMI study [1] have shown an increased risk for VF in patients with AF [4]. AF at initial presentation in the context of acute MI has also been associated with an increased risk of in-hospital VF in the FAST-MI 2005 registry [3]. Furthermore, a large Dutch population-based case-control study (the ARREST study) found similar increases in the risk of out-of-hospital VF arrest associated with a preceding diagnosis of AF [2]. In this study, 1397 out-of-hospital VF cases were compared with 3474 age- and sex-matched controls from the general population (i.e., without cardiac arrest). AF was associated with a threefold increased risk of VF (OR = 3.1, 95% CI 2.1–4.5). Lastly, in a Danish cohort AF has been reported to cause an increased risk of SCD following acute myocardial infarction [6].

Also several genetic studies have supported the idea behind the hypothesis of this study. The first GWAS performed on AF identified an association of SNP rs2200733 on chromosome 4q25 with AF in those of European and Asian descent [27]. Since this important finding, several studies have found the same association in other populations and also with an increased risk for prolonged PR interval, [28] which has been shown to be associated with all-cause mortality [29]. In a meta-analysis involving 716 SCD cases, the rs2200733 variant in 4q25 was found to be significantly associated with SCD (relative risk (RR) of 1.28 per minor T allele (95% CI: 1.11–1.48; *P* = 7.9 × 10^−4^) [15]. The latter study supports a joint genetic pathway between AF and VF or at least abnormal cardiac function. The rs2200733 has been associated with expression of *PITX2*, which is a transcription factor, and the expression level of *PITX2* has been associated with structural changes as well as expression of ion channel genes such as *SCN5A* [30]. Furthermore, the SNP rs6795970 which is in high linkage disequilibrium (r^2^ = 0.93) with rs6801957 at the *SCN10A* locus has previously been associated with abnormality of cardiac conduction [16, 17, 23]. The SNP rs6795970 has also been associated with AF in two different studies [23, 24]. Furthermore, Chambers et al. identified that rs6795970 is associated with prolonged cardiac conduction (longer P-wave duration, PR interval and QRS duration) [17]. In contrast, in the same study [17] the rs6795970 was significantly protective against risk of VF caused by first STEMI among the 976 participants in the **A**rrhythmia **G**enetics in The **NE**therlands (AGNES) study with an OR of 0.79 (95% CI: 0.66–0.95; *p* = 0.01). In the GEVAMI cohort we were not able to show an association between the SNP rs6795970 and VF. Obviously, since the A risk allele did not exist in the current GEVAMI population. This could be due to the selected STEMI population, and according to our knowledge the frequency of this SNP is not reported in a general Danish population. However, as a proxy for the SNP rs6795970, we previously published another SNP (rs10428132) which is also located near the *SCN10A* gene at chromosome 3, and which is in highly linkage with SNP rs6795970 (r^2^ = 0.966) and found no association with VF [20]. However, the mechanisms linking genetic variants in *SCN10A* with cardiac conduction remains to be determined. As mentioned earlier several studies have found rare and comment variants associated with AF. However the present AF-data only account for a very limited percentage of the heritability of AF and this is also the case for SCD and VF caused by STEMI. Therefore, identification of additional AF and VF loci by new technologies such as next generation sequencing may improve our knowledge of heritability of AF and VF and thereby understanding of if a shared genetic mechanism exists between the two arrhythmias.

There is also evidence for the proarrhythmic feature of AF, which by itself can induce VF without genetic involvment [31]. One study suggested that rapid ventricular rate during AF will reduce the ventricular refractoriness and induce ventricular tachyarrhythmia [32]. Moreover, the irregular rhythm of AF may cause VF through abrupt short-to-long changes in the cycle length, [33] and in AF patients with an implantable cardioverter defibrillator have more frequently and significantly induced VT/VF [34]. Lastly, AF by hemodynamic changes, can decrease parasympathetic tone and increase sympathetic tone making emerging of VF more likely [35].

The strength of the GEVAMI study is the prospective design and the use of a well-defined phenotype (VF). It is possible that some of these SNPs would have reached significance in a larger sample. In current study, the absolute number of patients with AF was low. A total of 16 (7%) cases and 10 (2%) controls were known with previous AF. Given our relatively low sample size, we acknowledge that this study may has been underpowered to detect associations. Most AF SNPs are high-frequency variants with small incremental effects on risk and therefore may limit the ability of this study to find association of AF-variants with small effect size. It is also important to mention that collecting cases is very time consuming and limited due to relatively low incidence of VF and difficulties to enroll and collect blood samples in STEMI-patients with VF and cardiac arrest. This is mostly due to that patients who died outside of the hospital or died in-hospital prior to enrollment cannot be included. The association of AF and VF in the cohort studies may be due to that AF patients have more comorbidity and therefore develop VF during acute ischemia. We only included patients with VF within 12 h before PPCI, therefore our data only represent patients with VF within this time interval since patients with late VF (VF after 12 h, or VF during PPCI) were excluded. Finally, our results in this population with white, European ancestry may not be generalizable to other populations.

## Conclusion

In this study we found that the 24AF-associated SNPs may not be involved in increasing the risk for VF. The complexity and interconnections between the two arrhythmias needs to be investigated in larger aspects and not only based on SNPs. Larger VF cohorts and use of new next generation sequencing and epigenetic may in future identify additional AF and VF risk loci and improve our understanding of genetic pathways behind the two arrhythmias.

## Abbreviations

AF: Atrial fibrillatiom
GEVAMI: GEnetic causes of Ventricular Arrhythmias in patients with first ST-elevation Myocardial Infarction
GWAS: Genome-wide association studies
IQR: Interquartile range
OR: Odds ratio
PPCI: Primary percutaneous coronary intervention
SCD: Sudden cardiac death
SNP: Single nucleotide polymorphism
STEMI: ST-segment elevation myocardial infarction
VF: Ventricular fibrillation
